# Canadian Adjuvant Initiative Workshop, March 26–27, 2013—Ottawa, Canada

**DOI:** 10.4161/hv.26972

**Published:** 2013-11-05

**Authors:** Lakshmi Krishnan, Susan Twine, Volker Gerdts, Luis Barreto, James C Richards

**Affiliations:** 1 National Research Council-Human Health Therapeutics (NRC-HHT); Ottawa, ON Canada; 2 Vaccine and Infectious Disease Organization-International Vaccine Centre (VIDO-InterVac); Saskatoon, SK Canada

**Keywords:** vaccines, adjuvants, vaccine delivery systems, bioprocessing, veterinary vaccines, regulatory approval

## Abstract

Novel adjuvants hold the promise for developing effective modern subunit vaccines capable of appropriately modulating the immune response against challenging diseases such as those caused by chronic and/or intracellular pathogens and cancer. Over the past decade there has been intensive research into discovering new adjuvants, however, their translation into routine clinical use is lagging. To stimulate discussion and identify opportunities for networking and collaboration among various stakeholders, a Canadian Adjuvant Initiative Workshop was held in Ottawa. Sponsored by the National Research Council Canada, Canadian Institutes of Health Research and the Vaccine Industry Committee, a two day workshop was held that brought together key Canadian and international stakeholders in adjuvant research from industry, academia and government. To discover innovation gaps and unmet needs, the presentations covered a board range of topics in adjuvant development; criteria for selection of lead adjuvant candidates from an industry perspective, discovery research across Canada, bioprocessing needs and challenges, veterinary vaccines, Canadian vaccine trial capabilities, the Canadian regulatory framework and WHO formulation laboratory experience. The workshop concluded with a discussion on the opportunity to create a Canadian Adjuvant Development Network. This report details the key discussion points and steps forward identified for facilitating adjuvant development research in Canada.

## Introduction

Vaccines are one of the cost effective contributors to improvements in public health and reduction in morbidity and mortality. New advances in vaccinology continue to improve our ability for rational design of efficacious vaccines against emerging and re-emerging infectious and chronic diseases.^1^ This includes rapid growth in diverse areas from genomics to nanotechnology that offer novel insights for immunogen design, adjuvant discovery and vaccine formulation. In particular, adjuvants, immunomodulatory agents used in conjunction with vaccines to enhance the immunity of antigens are largely recognized as the solution for directing antigen-specific immune responses of the appropriate quality, quantity, and durability to tackle evasive and/or chronic pathogens.^2^ Adjuvants also hold promise for vaccine dose sparing, improving immunity in the immunocompromised population (such as elderly, young children or individuals with other chronic co-morbidities), and for overcoming immune check-point responses in chronic disease that naturally mute immunity.^3^ The last decade has seen a burgeoning of research on adjuvant discovery. However, translation of adjuvant research into successful vaccines remains challenged by many factors, including lack of understanding of mechanism of action in humans, safety/reactogenicity profile, and effective bioformulation. A coordinated approach from bench to bed-side is required to fully benefit from the translational potential of novel adjuvant discovery research.

Recognizing this need, recently a Canadian Adjuvant Network Initiative workshop was held at Ottawa (March 26–27, 2013). The two day interactive meeting brought together leading opinion leaders from Canadian and global stakeholders in vaccine and adjuvant research; academic and government researchers, clinicians, industry representatives, policy makers, and funding agencies (Table 1, participant list). The current state of the art in adjuvant discovery research was reviewed, and participants discussed the opportunities and challenges for a coordinated approach to develop new and improved adjuvants for various vaccines. It was envisaged that a Canadian consortium for knowledge and best practices sharing among various stakeholders could facilitate the bench to bedside development of novel adjuvants.

**Table:** **Table 1.** Participant List for Canadian Adjuvant Initiative Workshop: March 26–27, 2013, Ottawa

**National Research Council** Dr Jim Richards Dr Lakshmi Krishnan Dr Wayne Conlan Dr Andrew Cox Dr Wangxue Chen Dr Amine Kamen Dr Michel Gilbert Dr Sue Twine Dr John Balsevich Mr Stacey Nunes Dr Luis Barreto	**Canadian Institutes of Health Research** Dr Marc Ouellette	**Industry Canada** Mr Philip Jennings Mr Mark Schaan Ms Reena Lutchmun
**Canadian Food Inspection Agency** Dr Anil Nichani	**Public Health Agencies of Canada** Dr Rainer Engelhardt Dr Frank Plummer Dr Gary Kobinger Dr John Spika Dr Ken Scott Dr Barb Law Dr Barb Raymond	**Health Sciences North/AMRIC** Dr Francisco Diaz-Mitoma
**Laval University** Dr Denis Leclerc	**University of Lausanne** Dr Patrice Dubois	**Queen’s University** Dr Sam Basta
**Dalhousie University** Dr Scott Halperin	**McGill University** Dr Brian Ward	**World Health Organization** Dr Martin Friede
**Health Canada** Dr Maria Baca-Estrada	**BC Centres for Disease Control** Dr Karuna Karunakaran	**University of British Columbia** Dr Bob Hancock
**Vaccine Industry Committee of BIOTECanada and RX&D** Dr Ron Boch (BIOTECanada) Dr David Burt (GSK) Dr Nathalie Garçon (GSK) Dr Heather Davis (Pfizer) Dr Terry Gunter (Pfizer) Dr Tony D’Amore (Sanofi Pasteur) Dr Nausheen Rahman (Sanofi Pasteur) Dr Rob Van Exan (Sanofi Pasteur) Dr Frédéric Ors (Medicago) Dr Nathalie Charland (Medicago)	**BC Children’s Hospital** Dr Tobias Kollmann	**University of Saskatchewan** **Vaccine and Infectious Disease Organization- International** **Vaccine Centre** Dr Andrew Potter Dr Volker Gerdts Dr Yan Zhou

The workshop was sponsored by the National Research Council of Canada (NRC), Canadian Institute for Health Research (CIHR), and the Canadian Vaccine Industry Committee (VIC). The workshop opened with introductory remarks from key speakers; Mr Philip Jennings (Assistant Deputy Minister, Industry Canada), Dr Jim Richards (Director, Vaccine Program, NRC), Dr Marc Ouellette (Scientific Director, CIHR-Institute of Infection and Immunity), Dr Rainer Englehart (Assistant Deputy Minister, Public Health Agency Canada), and Dr Fred Ors (Second Vice Chair, VIC). In his opening remarks, Mr Philip Jennings, noted Canada’s foundational strength in vaccines R&D conveying that “the federal government supports the growth of the Canadian vaccines cluster of expertise and the adjuvant sub-sector through continued investment and policy development.” Dr Richards gave an overview of NRC as a Research and Technology organization that had recently consolidated its research activities in the area of Vaccine development into a National program, with a mandate to actively collaborate with the industry and other stakeholders to rapidly bring new vaccines to market. Dr Ouellette shared the CIHR vision of Vaccines for the 21st Century, with almost $3 million invested in adjuvant research. Collectively the speakers of the opening session acknowledged the gap in coordination and translation of adjuvant research. However, it was noted that Canada has a history of early adoption of new vaccine technologies for public benefit. A prominent example is the Connaught Research Laboratories (now Sanofi Pasteur) established in the 1900s which was instrumental in the control of Diphtheria and Polio epidemics. Most recently, Canada took a leadership role in licensing the adjuvanted pandemic H1N1 influenza vaccine in 2009. The opening session concluded with participants being encouraged to develop an action plan for facilitating development of the Canadian adjuvant development network.

## Adjuvant Development

In the next session entitled “Industry perspectives on development and commercialization of adjuvanted vaccines/biotherapeutics,” representatives from the industry provided a perspective of the challenges for the development of novel adjuvanted vaccines. Dr David Burt from Glaxo Smith Kline (GSK) presented on the topic of “From MPL to Cervarix: Challenges in taking adjuvants and adjuvanted vaccines from discovery to commercialization.” GSK has spear-headed introduction of novel adjuvants in commercial vaccines. Optimum formulation of adjuvant and vaccine is critical for maximizing efficacy and ease of use. The development of monophosphoryl Lipid A and its use in combination with aluminum hydroxide to form the ASO4 adjuvant system is a vaccine success story that represents the effective use of combination-adjuvant formulations for minimizing reactogenicity and maximizing efficacy. Key consideration in the development process was also the ability to scale-up the consistent production of synthetic analogs of monophosphoryl lipid A. The AS04 adjuvant, in combination with the Cervarix^®^ vaccine resulted in elicitation of long-term high levels of neutralizing antibodies, against human papilloma virus (causative agent of cervical cancer) and 4.5 million doses have been administered in the United Kingdom with an acceptable safety profile.

Dr Heather Davis (Pfizer) presented a case study of the CpG oligonucleotide (ODN) adjuvant development. The adjuvant activity of CpG ODNs was discovered nearly 2 decades ago; stimulation of TLR9-dependent innate immune responses. This was an academic research discovery that resulted in the spin-off company of Coley Pharmaceutical Group. Early on, licensing opportunities were explored with various pharmaceutical companies for the use of CpG as a vaccine adjuvant, and generating human clinical data was necessary to attract the interest of Big Pharma companies. Moreover, even with good supporting data from human studies, various other considerations influenced the licensing and further development of CpG adjuvants. The economic environment, real or perceived safety considerations, ease of manufacturing and formulation, and differing priorities of big pharma can all impede licensing and development of new and promising technologies. Another important consideration is time. Thus, CpG ODN adjuvant has still not found use in a commercial vaccine. Overall the development path for adjuvanted vaccines is complex and convoluted and can take >2 decades. Thus, Dr Davis pointed out that it was important to consider potential road-blocks for commercialization and de-risk the technology early-on during research development process.

Finally, Dr Nathalie Garcon (GSK) presented an overview of adjuvant development research and use with commercial vaccines. The first vaccine adjuvant that was licensed for human use was alum in 1924. For the next 6 decades, no new adjuvants were introduced, until in 1997, MF59 produced by Novartis was approved for use with a flu vaccine in Europe. ASO4 comprising alum and monophosphoryl lipid A was licensed with Cervarix^®^ in 2005 by GSK. Interestingly, the complete mechanism of action of alum is yet to be revealed. Indeed one could consider that if alum was discovered today, it would need further evaluation before “universal” use with all vaccines. Adjuvant research has burgeoned in the last two decades fuelled by the quest to develop vaccines against evasive pathogens such as HIV that required potent induction of immune responses. Nevertheless, it is important to recognize that there should be a clear value-added benefit for use of an adjuvant with a given vaccine. Currently, there are many adjuvants in development and at GSK, the lead candidates being evaluated include ASO1, ASO3, ASO4, and ASO15.^4^ These adjuvant systems are comprised of multiple components; immunostimulatory and stabilizing vaccine carrier systems. For example, ASO15 is a liposome based adjuvant system comprising monophosphoryl lipid A, QS21, and CpG ODN. Vaccine development is a multi-decade long process; for example the adjuvant potential of monophosphoryl lipid A and QS21 was described in published literature in the mid-1980s. Three decades later, GSK is aiming to develop a whole platform of adjuvanted vaccines with these novel compounds. Dr Garcon pointed out that when companies reactively seek collaborations on new adjuvant technologies, it is often to bridge a gap in a specific arm of the immune response that needs to be induced for which current adjuvants are not sufficient. However, sometimes, a promising technology may be evaluated for proof of concept with several vaccines. At GSK, the adjuvant needs across the organization are harmonized and evaluated centrally. GSK also has made ~50 supply agreements in the lasts 10 y for their in-house portfolio of adjuvants for vaccine products with external clients. Various collaborative models including material transfer agreements are considered and executed as appropriate to meet the business need of the involved organizations, to protect intellectual property, quality of study, and outcomes.

Overall this session highlighted the business perspective on adjuvant development. There are many key sequential steps in preclinical adjuvant development such as: (1) designing and defining an adjuvant based on the type of immune response to be induced; (2) understanding the host pathogen response and selecting an appropriate antigen and adjuvant to augment the necessary protective immune response; (3) consistent, high purity production of the adjuvant and antigen; (4) developing and qualifying immune readout assays; (5) optimizing the vaccine formulation ensuring compatibility between antigen and adjuvant to retain the potency of each; (6) pre-clinical evaluation; and (7) preclinical toxicology and safety. Each of these key steps should be performed in such a way that they can predict the outcome of the phase I clinical trial. Moving toward phase I clinical trial requires defined processes for GMP production for the antigen, adjuvant, and ultimately the formulation. Phase I clinical trials are designed to provide information on safety and immunogenicity profile of the selected vaccine product. Phase II clinical trials will be designed to expand upon evaluation of safety, antigen, and adjuvant dose and also the correlates of protection. Finally, phase III efficacy studies are designed to evaluate the vaccines in >10 000 subjects. Usually, at this point small biotech companies developing vaccines will need to partner with Big Pharma companies to undertake such extensive and costly evaluation of a vaccine. It must also be appreciated that post-licensure the evaluation of various parameters of vaccine safety and efficacy continues for the entire lifetime of the product on the market. Overall, when developing an adjuvant it is important to recognize that the formulation of the adjuvant and antigen is the ultimate product and so the process should result in a simple, stable, safe and efficacious product.

## Adjuvant Landscape in Canada

This session highlighted that Canadian researchers have been at the forefront of novel adjuvant discovery. Dr Bob Hancock (University of British Columbia [UBC]) highlighted how a collaborative project involving multiple nodes of researchers in Canada was funded by the Gates Grand Challenges in Global Health Initiative and supported the development of a novel combination adjuvant system. The project goal was to develop a platform for single dose vaccine in neonates and help overcome the technological barriers for health delivery in developing countries against multi-drug resistant microorganisms. First, a multidisciplinary high throughput screening effort at UBC queried ~200 000 innate immune interactions to identify potent host defense peptide adjuvants. Second, collaborating with scientists from the Vaccine and Infectious Disease Research Organization (VIDO)-InterVac (International Vaccine Centre), a combination adjuvant system was developed for potent induction of immunity to a pertussis vaccine after single dose systemic application. This triple adjuvant system comprised host defense peptides for augmenting potent innate immune stimulation, polyphosphazenes for delivery, and CpG ODN/poly IC for activation and durability of the response.^5^ This unique adjuvant system offered many advantages; 100–1000 fold increase in potency of a pertussis vaccine, consequent antigen dose sparing, efficacy in neonate models, versatile compatibility with varied antigens (DNA, peptide, protein, live attenuated), balanced Th1/Th2 immunity, and lack of adverse events. Dr Volker Gerdts of VIDO-InterVac further described the efficacy of this adjuvant system in various animal models; mice, pigs, cattle, and cotton rats. Besides pertussis, this adjuvant system has been evaluated with a Respiratory Syncytial Virus (RSV) vaccine candidate in the cotton rat model, and demonstrated to be highly effective for mucosal immunization.^6^^,^^7^

NRC has a portfolio of novel adjuvants under development. First, Dr Lakshmi Krishnan presented on archaeosomes which are an archaeal liposomal delivery system with potent adjuvant activity for induction of systemic cell-mediated immunity including CD8^+^ T cell responses.^8^ This vesicular delivery system is constituted of highly stable archaeal lipids with unique immunomodulating properties augmenting dendritic cell activation and co-stimulation in the absence of overt inflammation. Additionally, archaeosomes act naturally as pH-sensitive liposomes delivering the antigen cargo into the cytosol following phagosomal acidification leading to potent induction of CD8^+^ T-cell responses. Proof of concept studies with an anti-listeria and anti-melanoma vaccine in animal models has demonstrated durability of the immune response.^9^^,^^10^ More recently, in-keeping with the strategy for easy scale-up, a semi-synthetic approach for formulating archaeosomes comprised of defined lipids has been effectively adopted.^11^ Dr Wangxue Chen highlighted that archaeal lipids may be constituted with divalent cations for example calcium ions into AMVAD (Archaeal Lipid Mucosal Vaccine Adjuvant Delivery system) for effective mucosal delivery. Intranasal administration of AMVAD evokes secretory IgA as well as systemic IgG responses against a tularemia vaccine in mice.^12^ Archaeal lipids have also been shown to lack toxicity and reactogenicity in animal models even after repeated administration.^13^ Furthermore, to aid commercialization of the archaeosome technology, NRC has invested in building a strong intellectual property portfolio and scale-up processes for archaeal lipid production. Dr John Balsevich indicated that NRC is also developing novel saponin-based adjuvants, with unique structures, low hemolytic activity, and strong adjuvanticity that can be produced indigenously by Canadian plants in large quantities, and obtained as single species at high purity. Additionally, novel recombinant *Salmonella* bacterial vector based technology is being developed by NRC.^14^

A novel hypothesis driven approach to adjuvant discovery was described by Dr Garry Kobinger (National Microbiology Laboratory, PHAC). He hypothesized that the breadth and amplitude of immune response can be related to how frequently a specific amino acid sequence is found in nature. Thus, short (3–5 amino acids) random peptides were synthesized and screened for immunomodulatory activity. A 5-mer non-natural sequence was identified to possess adjuvant potential, and tested for its ability to improve the immunogenicity of commercial Hepatitis B vaccine in mice. Further studies indicated that this 5-mer sequence augmented NK cell activation which in turn directed adaptive immunity in the absence of inflammation. This adjuvant system is now being evaluated in primate model for a viral vaccine.^15^

Dr Denis Leclerc presented the adjuvant platform being developed by Folia Biotech a Canadian start-up operational since 2008. A nano virus-like particle (50–100 nm) based adjuvant is comprised of papaya mosaic virus protein, and a TLR 7/8 agonist RNA sequence. An influenza vaccine formulation comprising this high stable adjuvant triggered higher Haemaglutinin titers in the lungs of vaccinated mice relative to the non-adjuanted vaccine after intranasal administration.^16^ Furthermore toxicity studies were performed in rabbits and rats and demonstrated complete biodegradability of the formulation after 72 h. The adjuvanted influenza vaccine is now being advanced to clinical trial, and cGMP lots are being produced. Upon discussion with Canadian regulators, Folio Biotech is designing the phase I trial to include the vaccine and also an adjuvant-alone arm for evaluation. This latter arm of the study will evaluate adjuvant safety independently of the antigen and is a de-risking strategy for novel adjuvants.

Dr Sam Basta (Queen’s University) highlighted the need to rationally design vaccines capable of evoking CD8^+^ T-cell response particularly for emerging and re-emerging vaccines. He noted that currently the only approach for consistent induction of CD8^+^ T-cell responses are attenuated live vector delivery systems. However, it is possible to harness the potential of cross-presentation, wherein the antigen presenting cells uptakes particulate debris (such as a dying cell) and cross-presents antigen to CD8^+^ T cells.^17^ Nevertheless, the optimal nature of antigen required for efficient cross-presentation is still unclear. Identification of adjuvants that can facilitate cross presentation will be of advantage. Another key consideration is the immunodominance hierarchy particularly when developing multi-valent vaccines.^18^

## Bioprocess and Formulation

Scientists from Sanofi Pasteur, Drs Tony D’Amore and Nausheen Rahman shared some of their challenges in vaccine bioprocessing and formulation and adjuvant research, in the context of the 13 vaccines currently in their development portfolio. A particular challenge was indications that are more complex and difficult to test, that may require use of new adjuvants. Use of novel compounds and formulation technologies require additional scrutiny and validation and can slow vaccine development due to significant challenges in vaccine regulation.

In terms of vaccine formulation, stability was a key consideration for vaccine manufactures, with the desire to develop both liquid and dry adjuvant formulations that will facilitate widespread field usage. Bioprocess R&D at Sanofi Pasteur is focused upon manufacturing process to provide material for clinical trial experimentation, with upstream, downstream, and formulation innovations. Capabilities include a large expertise in formulation and design, adjuvant development and stability, and process monitoring. Highlights of challenges on the horizon include identifying a wider toolbox of adjuvants, and understanding the mechanism of action of adjuvants. For ease of manufacturing, bioprocessing and scale up considerations become important while choosing adjuvants and qualifying mixing dynamics. The company frequently works with partners, scanning for new opportunities in adjuvants and immunomodulators. A recent collaboration with VaxDesign was highlighted, with a vision to accelerate clinical trial development, decreasing the time to market for vaccines. The in vitro high throughput microtiter plate-based screening system from VaxDesign mimics the diversity of the human immune system by using cells from varied donors that are selected for age, gender etc. to evaluate the effectiveness of vaccines. The technology aims to simulate human clinical vaccine trials without the need for human subjects. This can afford advantages of speed, cost, and ethical approval if validated for use in vaccine development. Thus, the vaccine industry can be supported by new innovative technologies and in-vitro assay systems for testing efficacy and mechanism of action of vaccines and adjuvants.

Dr Patrice Dubois described the activities of the Vaccine Formulation Laboratory at the University of Lausanne, Switzerland. This laboratory is a WHO collaborating centre and was founded in 2010, with the mandate to transfer adjuvant technology and provide training and assistance to users with vaccine formulation.^19^ The primary users of this service are the public sector, small biotechnology companies and developing country vaccine manufacturers. The choice of adjuvant is focused on mature technologies with a demonstrated safety profile in human trials, particularly aluminium salts, oil in water emulsions, water in oil emulsions, saponins, and TLR agonists. All processes for vaccine formulation are tailored for ultimate use in a Good Manufacturing Practices (GMP) environment. A case study was highlighted in which technology transfer for an adjuvant formulation was effectively facilitated to a Biofarma, an Indonesian vaccine manufacturer. This vaccine manufacturer was the first technology transfer recipient of the Vaccine Formulation Laboratory, in a U.S. Department Human Health Services Biomedical Advanced Research and Development Authority (US HHS BARDA) and GIZ (German International Cooperation Agency) funded project to build up developing countries vaccine manufacturers adjuvant production capabilities for pandemic influenza. This project included support for on-site installation of equipment, vaccine production, and establishment of preclinical studies.^20^ A similar project involved the Vietnam’s Institute of Vaccine and Medical Biologicals (IVAC). As part of the European Union (EU)-funded TRANSVAC project which aimed at providing trans-national access to vaccine technologies, the Vaccine formulation laboratory also provided access to formulation services to European recipients. In the context of TRANSVAC, the laboratory held vaccine development training courses covering the whole spectrum of activities involved in bringing vaccine candidates from bench to clinical trials. Two one-week courses were held at the University of Lausanne in September 2012 and March 2013. Overall the Vaccine Formulation Laboratory facilitates the translation of know-how from bench to industry and clinical testing.

## Veterinary Vaccines and Animal Models of Human Diseases

Often, early immunogenicity studies during vaccine development are restricted to mice models. However this may limit discovery of novel adjuvants or fail to predict potential adverse effects. Mice models may not always predict quality and quantity of immune responses in humans. Use of other animal models, particularly large animal models such as pig can provide a valuable choice for comprehensive evaluation of vaccines. Drs Volker Gerdts and Andrew Potter highlighted the capabilities of VIDO-InterVac in Canada which is recognized as a global leader in the use of large animal models for vaccine development research. Dr Potter pointed out that ~50% of all diseases are recognized to have zoonotic transmission. As animal species can serve as reservoirs for transmission of many pathogens, curtailing spread of the pathogen by animal vaccination (so called food safety vaccines) can be an effective strategy for management of human disease. In partnership with Bioniche and the Alberta Livestock and Meat Agency VIDO-InterVac, and the University of British Columbia developed the world’s first *E.coli* O:157 vaccine.^21^ VIDO-InterVac was created in 1975 in Saskatoon, Canada as a joint venture between two provincial governments; Alberta and Saskatchewan. Since its inception, VIDO-InterVac has a history of translating vaccine discoveries in human and animal health. Currently as part of the University of Saskatoon, VIDO-InterVac is a financially independent self-sustaining research institution. Canada’s largest BSL3 facility and one of the most advanced facilities in the world, the International Vaccine Centre (InterVac; www.vido.org), was recently certified to enhance VIDO-InterVac’s capacity to work with Biosafety Level 3 (BSL3) pathogens.

Another Canadian success story is PREVENT (Pan-Provincial Vaccine Enterprise Inc.) established in 2008 as Centre of Excellence in Commercialization and Research with funding from the Canadian federal government. PREVENT was designed to fill a gap in clinical development for promising Canadian vaccine research. As a not-for-profit commercial entity, PREVENT focuses on risk reduction for promising technologies in the early clinical development stage. PREVENT supports technologies after careful evaluation of the intellectual property portfolio and competitive landscape for late stage research technologies. A number of Canadian technologies have moved closer to advanced clinical trials with PREVENT support including an ALS vaccine, Chlamydia vaccine, and Folio Biotech’s adjuvant platform.

## Vaccine Trial Capabilities in Canada

Dr Scott Halperin (Dalhousie University) gave an overview of the networks of vaccine research within Canada. Dr Halperin began by describing the Canadian Association for Immunization Research and Evaluation (CAIRE). The network comprises investigators from academia and public health with the combined mandate of enhancing vaccinology research in Canada, to ensure that Canadians have timely access to new vaccines and vaccine programs.^22^ In addition, CAIRE has organized a Research Sponsor Advisory Board, with representatives from vaccine manufacturers, aimed at exploring joint interests.

The Canadian Immunization Monitoring Program ACTive (IMPACT) was also described. IMPACT is a pediatric hospital-based national active surveillance network for adverse events following immunization, vaccine failures in children. This is particularly focused upon diseases that are, or soon will be vaccine preventable. This network extends across 12 Canadian centers and reports adverse events that are temporally associated with vaccination. Observations are reported for follow up to local public health agencies.

Pandemic influenza research network (PCIRN) was established in 2009 in response to anticipated influenza pandemic, funded by the Canadian Public Health Agency. Originally aimed at developing procedures to evaluate pandemic influenza vaccine, the network brings together Canada-wide expertise in vaccine evaluation. This serves to increase Canada’s capacity to rapidly test influenza vaccine candidates, and facilitate knowledge exchange.

Dr Halperin also reviewed the Canadian vaccine clinical trials capacity across the country including the Vaccine Evaluation Center in Vancouver, sites at the University of Alberta and University of Calgary, sites in Winnipeg and Ontario, the Vaccine Study Centre at McGill University, and INSPQ (Institut national de santé publique) in Quebec City, as well as the Canadian Center for Vaccinology (CCfV). CCfV headed up by Dr Halperin is a collaborative effort with Dalhousie University, the IWK (Izaak Walton Killam) Health Centre, and Capital Health. The CCfV comprises a multi-disciplinary research team aimed at advancing vaccine research and translation, with expertise ranging from microbial research to clinical trials. A unique to Canada 10-bed human vaccine challenge unit provides leading edge capacity and is one of only a dozen such facilities world-wide.

## Regulatory Framework for Adjuvanted Vaccines

In Canada, vaccines are regulated by the Biologics and Genetic Therapeutics Directorate (BGTD) of Health Canada. Dr Maria Baca-Estrada (BGTD) presented an overview of the regulatory framework for adjuvanted vaccines. She highlighted the need to engage in discussion with regulatory agencies early on during the adjuvant development process, to understand the key steps in the development process that will impact regulatory approval. Adjuvants are not approved as a stand-alone product but as part of the vaccine formulation. Therefore, it is important to demonstrate as part of the nonclinical assessment the need to include the adjuvant such as the increased magnitude and/or breadth of the immune response, dose sparing effects, etc. Dr Martin Friede of the WHO added to the discussion emphasizing that use of adjuvant with each antigen is independently evaluated, and continues for the lifetime of the product. Consistency in manufacturing, characterization of lots, stability, and shelf-life should all be well documented. WHO in collaboration with various regulatory agencies has released a recent guideline on adjuvant use for vaccines (http://www.who.int/biologicals/expert_committee/en/) which is soon expected to be endorsed by an International expert committee.

In Canada, Veterinary biologics including vaccines are regulated by the Canadian Food Inspection Agency (CFIA). Dr Anil Nichani provided an overview of the category of products regulated by CFIA. Veterinary use class I products are low-risk conventional vaccines, whereas class II products include modified vaccines such as those based on recombinant vectors, live organisms etc. In addition to efficacy, other consideration for licensing of veterinary vaccines includes cost effectiveness, ease of administration, ease of metabolism, and minimal effects of carcass in food animals. Human safety is a major concern, with desired zero residual vaccine retention in the animal over time. Veterinary vaccines can often serve as an opportunity for evaluation of cost-effectiveness and efficacy of human vaccines.

## Opportunities to Create a Canadian Adjuvant Network

The last session of the workshop focused on a panel discussion aimed at evaluating how a Canadian adjuvant consortium should be structured. It was identified that for a Country like Canada with the aging population, there was a medical need to deliver cost-effective public health and accelerated vaccine development against targeted diseases is an attractive solution. There is substantial vaccine development and adjuvant research and infrastructure capacity within Canada. Thus, coordination of efforts will be beneficial to accelerate the pace of fundamental discoveries to clinical practice (Fig. 1).

**Figure:**
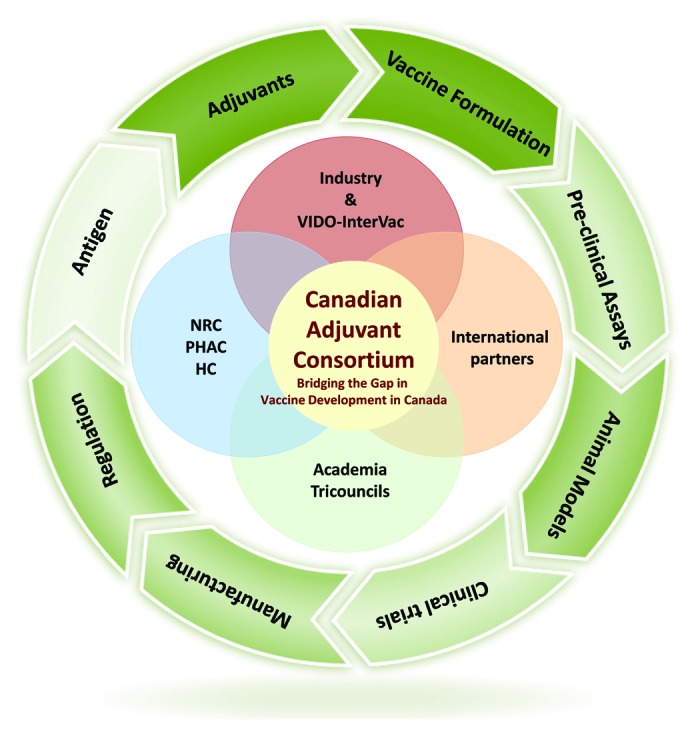
**Figure 1.** Canadian Adjuvant Consortium Concept. It is proposed that the Canadian Adjuvant Consortium can comprise a network of key stakeholder organizations: Government institutions and regulatory agencies (National Research Council of Canada [NRC], Public Health Agency of Canada [PHAC], Health Canada); Academia and Canadian Tri-council granting agencies (Canadian Institutes of Health Research [CIHR], National Science and Engineering Research Council [NSERC] and Social Sciences and Humanities Research Council [SSHRC]); Industry (Canadian Vaccine Industry Committee [VIC]), VIDO-InterVac (Vaccine and Infectious Disease Organization-International Vaccine Centre). The network should be structured to unite stakeholders through identification of common goals and funding opportunities to bridge the gap in vaccine development in Canada. The stakeholders bring competency in various areas from antigen and adjuvant discovery through regulation as schematically represented by the outer ring. These nodes of expertise and infrastructure can be leveraged through common consortium goals to hasten the translation of new Canadian adjuvant technologies and vaccine products from bench to bed-side.

Key steps in the formation of a Canadian consortium will include: (1) defining a unifying goal for all stakeholders, (2) defining the key activities that will be undertaken by the consortium, (3) identifying strategies for mitigating intellectual property risks through knowledge sharing, (4) mechanisms for increasing the visibility of adjuvant research within Canada, and (5) identifying sources of funding to sustain consortium activities. Lessons can be learnt from other adjuvant consortiums such as GADI, Adjunet (Box 1), and networks in France and Japan. A working group led by NRC will focus on defining the objectives and framework for the development of an Adjuvant Consortium in Canada.

Box 1. **The Global Adjuvant Development Initiative**

The Global Adjuvant Development Initiative (GADI) was created to promote access to adjuvant know-how and technologies for public sector vaccine developers and developing country manufacturers. GADI is housed at WHO and serves as a umbrella initiative to bring together a network of adjuvant providers and users with the following aims:

• To promote and facilitate access to adjuvants

• To promote access to information on adjuvants

• To promote technology transfer of adjuvants

• To facilitate evaluation of new adjuvants

http://gadi.bio-med.ch/cms/

**AdjuNet**

The adjuvants and the knowledge and training related to adjuvant use that is being generated through GADI are being made available to the public sector through a network of vaccine development institutes and laboratories with an interest in using adjuvants in their vaccine development program. This network, called AdjuNet, is being created to facilitate sharing of data gained with the adjuvants being developed by GADI, to minimize duplication of studies, and to ensure that vaccines that are developed using adjuvants provided by GADI are made available to developing countries at affordable prices.

http://www.who.int/phi/implementation/techtransfer_vaccines_adjuvants/en/index.html

The panel discussion also identified the key scientific opportunities for adjuvant research. In particular adjuvant technologies that can direct CD8 T-cell responses in humans was currently lacking. Many adjuvants that hold promise for induction of CD8 T-cell responses based on murine model studies fail when evaluated in human clinical trials. Thus, qualified immunological assays using human immune cells that can predict in vivo induction of CD8 immunity in humans is required. Adjuvants that are effective for stimulating immunity through alternate routes (ex., mucosal) other than systemic are also desired. Identifying biomarkers as correlates of vaccine immunity in humans is another area that needs increased research activity.

## Conclusions

Canada has a strong academic sector and continuum of early-stage research to commercialization history and presence in both human and animal vaccines. Global vaccine industry players such as Sanofi Pasteur and GSK have major vaccine R and D and manufacturing Canadian footprint. Canada is also at the forefront of vaccine regulation and public health policy assisting the quick licensing and use of safe new vaccines. For example, Canadian vaccine manufactures and regulators worked in synchrony to facilitate the timely introduction of the adjuvanted H1N1 influenza pandemic vaccine in 2009. Thus, Canada can take an active leadership in vaccine adjuvant research and translation to commercialization of vaccines that could reduce global morbidity and mortality to emerging and re-emerging infectious diseases.

## Notes

10.4161/hv.26972
